# The Prevalence of Beta-Blocker Use Among Medical Students at King Saud bin Abdulaziz University for Health Sciences in Jeddah, Saudi Arabia: A Cross-Sectional Study

**DOI:** 10.7759/cureus.11450

**Published:** 2020-11-11

**Authors:** Rawan Alkhatabi, Joud Alowfi, Layan Arshad, Muhammad A Khan

**Affiliations:** 1 Medical Education, College of Medicine, King Saud Bin Abdulaziz University for Health Sciences, Jeddah, SAU; 2 Epidemiology and Public Health, King Saud Bin Abdulaziz University for Health Sciences, Jeddah, SAU

**Keywords:** propranolol, saudi arabia, medical students, beta blockers

## Abstract

Objectives

The aim of this study was to evaluate the prevalence and patterns of beta-blocker usage among medical students. Reasons for the use and demographic factors influencing their consumption were also evaluated.

Methods

This was an observational cross-sectional study among medical students at King Saud bin Abdulaziz University for Health Sciences (KSAU-HS) Jeddah, Saudi Arabia. Medical students were surveyed between February and April of 2019 using an electronic self-administered questionnaire. The questionnaire had close-ended questions, 18 of which were about demographics and 17 about propranolol use.

Results

A total of 234 medical students participated in the study, of whom 14.5% (95% CI: 10.44-19.49) reported using propranolol. Fifth-year medical students comprised 50% of propranolol users. The prevalence of males using propranolol was lower compared to females. The most common reasons for using propranolol were anxiety relief and performance enhancement before the objective structured clinical exams (OSCEs; 70.6%) and before oral presentations (38.2%).

Conclusion

The prevalence of propranolol use among medical students in KSAU-HS Jeddah was low compared to other studies, with the highest reported use among fifth-year medical students. The main reasons for using propranolol were anxiety relief during OSCEs and performance enhancement for presentations. Efforts must be directed towards raising medical students' awareness of the risks of inappropriate beta-blocker use to decrease its use and avoid potential adverse effects of the medication.

## Introduction

Medical students are prone to stress and burnout due to increasing academic demands, financial struggles, and social issues [1,2]. These factors can affect academic performance negatively, leading to more distress [3]. Current literature demonstrates that there are higher rates of psychological stressors among medical students compared to the general population [4]. A study in Lithuania found that symptoms of anxiety were prevalent in 43% of medical students [5]. A meta-analysis conducted by Rotenstein et al. reported a prevalence of depressive symptoms and suicidal ideation of 27.2% and 11.1%, respectively [6]. Similarly, in a cross-sectional survey among senior medical students in New York, 71% of students met the criteria for burnout [7].

Non-selective beta-blockers are used to manage performance-only social anxiety disorder (SAD), of which propranolol (Inderal) is the most commonly prescribed [8,9]. Propranolol exerts its action by inhibiting sympathetic input to the cardiovascular system, decreasing heart rate, and contractility [10]. This makes propranolol effective in decreasing the physical symptoms of anxiety [10,11]. According to a study in Switzerland, 13.8% of participating university students reported using prescription drugs for neuroenhancement. The most frequently used prescription medications in the study were methylphenidate (4.1%), sedatives (2.7%), and beta-blockers (1.2%) [12]. 

University students experience high levels of stress during oral and written examinations, and agents like propranolol are commonly used among such populations [12]. A local study among medical and dental students in Saudi Arabia found that around 30% used propranolol, of which 48% self-prescribed the medication [13]. Self-prescription of beta-blockers makes users more predisposed to the established adverse effects such as hypotension, bradycardia, bronchospasms, and hypoglycemia due to the lack of proper evaluation and supervision [14]. The main objective of this study was to determine the prevalence of beta-blocker use among medical students at King Saud bin Abdulaziz University for Health Sciences (KSAU-HS), Jeddah, Saudi Arabia. A secondary objective was to determine the causes of beta-blocker use among the same population.

## Materials and methods

Recruitment of participants

This was an observational, cross-sectional study among medical students of KSAU-HS Jeddah. It was conducted from February 2019 to April 2019. All medical students had an equal opportunity to participate in the study, and there were no exclusion criteria. This includes medical students from basic years, which are second to fourth year, and clinical years, which are from fifth to sixth year. The data were collected using a non-probability convenience sampling technique. The calculated sample size was 255 students, and 234 responses were included in the study.

Questionnaire

Students were surveyed using an online self-administered English questionnaire developed by the researchers. After validating the questionnaire through face validity, content validity, and pilot testing, it was sent to eligible participants electronically through email and a messaging service application. It consisted of 36 multiple-choice questions divided into three sections. The first section inquired about demographic information and baseline characteristics of the participants such as age, gender, and academic level. The last question of the first section inquired about the use of propranolol, and based on the answer to this question, they were directed to either of the following sections. Section two inquired about whether they were offered or recommended to use propranolol. While section three inquired about the details about the use of propranolol, such as prescription, frequency, and side effects.

Data analysis

Data were collected and entered in a Microsoft Excel sheet, and then exported to the Statistical Package for the Social Sciences (SPSS version 20, IBM, Armonk, USA) for the statistical analysis. Qualitative variables were reported as frequencies and percentages, and quantitative variables, such as age, were represented as means and standard deviations. The Chi-square test was used to compare the association of beta-blocker use among gender subgroups. The results were considered statistically significant if p < 0.05, and were reported with a 95% confidence interval.

Ethics and consent to participate

This study was approved by the Institutional Review Board (IRB) of King Abdullah International Medical Research Center (KAIMRC) in the Western region (Approval: SP18/522/J). Additionally, a consent form, which included the aim and objectives of the study, was required prior to participation. Privacy and confidentiality were ensured during the collection and management of the data. No personal information, such as names or contact information, were collected.

## Results

General results

**Figure 1:**
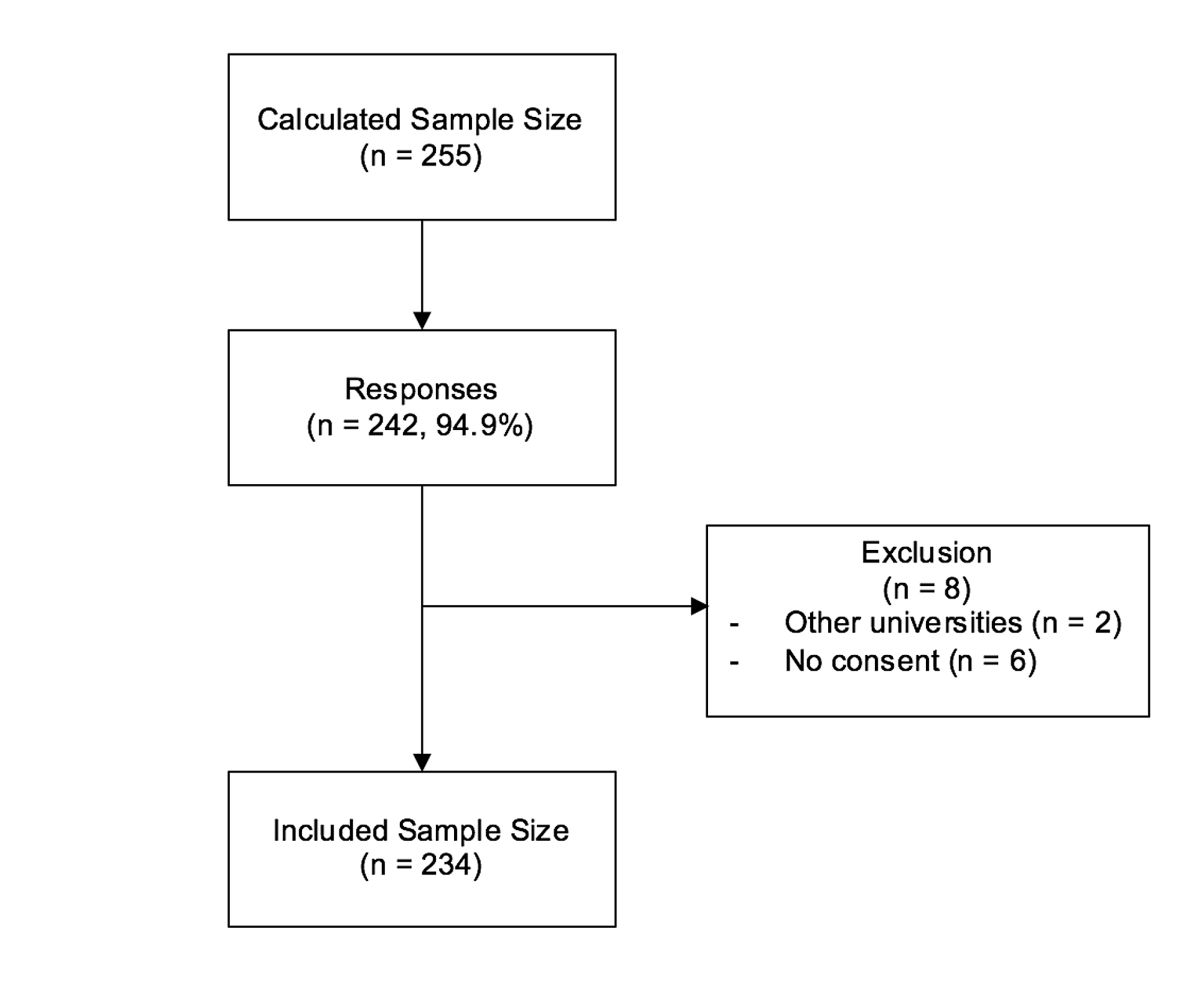
Participants Selection Criteria Flow Chart

The included sample size was 234 students who responded to the survey, with a response rate of 94.9% (Figure 1).

The demographic information and baseline characteristics are summarized in Table 1. Of the 234 participants, 34 (14.5%) reported using propranolol. The highest rate of propranolol use was among fifth-year medical students, representing 50% of the group (Table 1).

**Table 1:** Baseline Characteristics The baseline characteristics of the total 234 participants, and the characteristics of the 34 out of 234 participants who answered “Yes” to using propranolol. SD: standard deviation, GPA: grade point average. *Not the complete number of participants due to missing data.

Baseline Characteristics n (%)		
Characteristics	Propranolol users (n=34)	Total participants (n=234)
1- Gender		
Male	8 (23.5)	122 (52.1)
Female	26 (76.5)	112 (47.9)
Total	34 (100)	234 (100)
2- Age (Mean ± SD)	22.15 ± 1.96	22.39 ± 2.1
3- Academic year		
Third-year	11 (32.4)	64 (27.4)
Fourth-year	5 (14.7)	60 (25.6)
Fifth-year	17 (50)	90 (38.5)
Sixth- year	1 (2.9)	20 (8.5)
Total	34 (100)	234 (100)
4- GPA		
4.5 – 5	23 (67.6)	149 (63.7)
4.0 – 4.49	8 (23.5)	59 (25.2)
3.5 – 3.99	2 (5.9)	19 (8.1)
3.0 – 3.49	-	3 (1.3)
2.5 – 2.99	-	3 (1.3)
Total	33 (97.1)*	233 (99.6)*
5- Smokers	6 (17.6)	31 (13.2)
6- Use of psychoactive drugs	3 (8.8)	8 (3.4)
7- Use of energy drinks	10 (29.4)	45 (19.2)
8- Exercising	17 (50)	112 (47.9)
9- Chronic diseases	5 (14.7)	25 (10.7)
10- Allergies and asthma		
Allergies	7 (20.6)	48 (20.5)
Asthma	2 (5.9)	13 (5.6)
Both	2 (5.9)	9 (3.8)
Total	11 (32.4)	70 (29.9)
11- Diagnosed psychiatric disorder	10 (29.4)	31 (13.2)

Moreover, female students are 4.35 times more likely to be using propranolol as compared to males students (95% CI: 0.10-0.54, p<0.001; Table 2).

**Table 2:** Gender-Based Analysis Chi-square test. OR: odds ratio. *Significant value.

	Yes, n (%)	No, n (%)	Total	p-Value	OR (CI 95%)
Male	8 (6.6)	114 (93.4)	122	<0.001*	0.23 (0.10-0.54)
Female	26 (23.2)	86 (76.8)	112		
Total	34 (14.5)	200 (85.5)	234		

When inquired about smoking habits and the use of psychoactive drugs, six participants (17.6%) identified as smokers, and three (8.8%) reported using psychoactive drugs. Additionally, 10 participants (29.4%) were clinically diagnosed with a psychiatric disorder. Anxiety disorder was the most common illness reported (17.6%). 

Results of using propranolol

**Figure 2:**
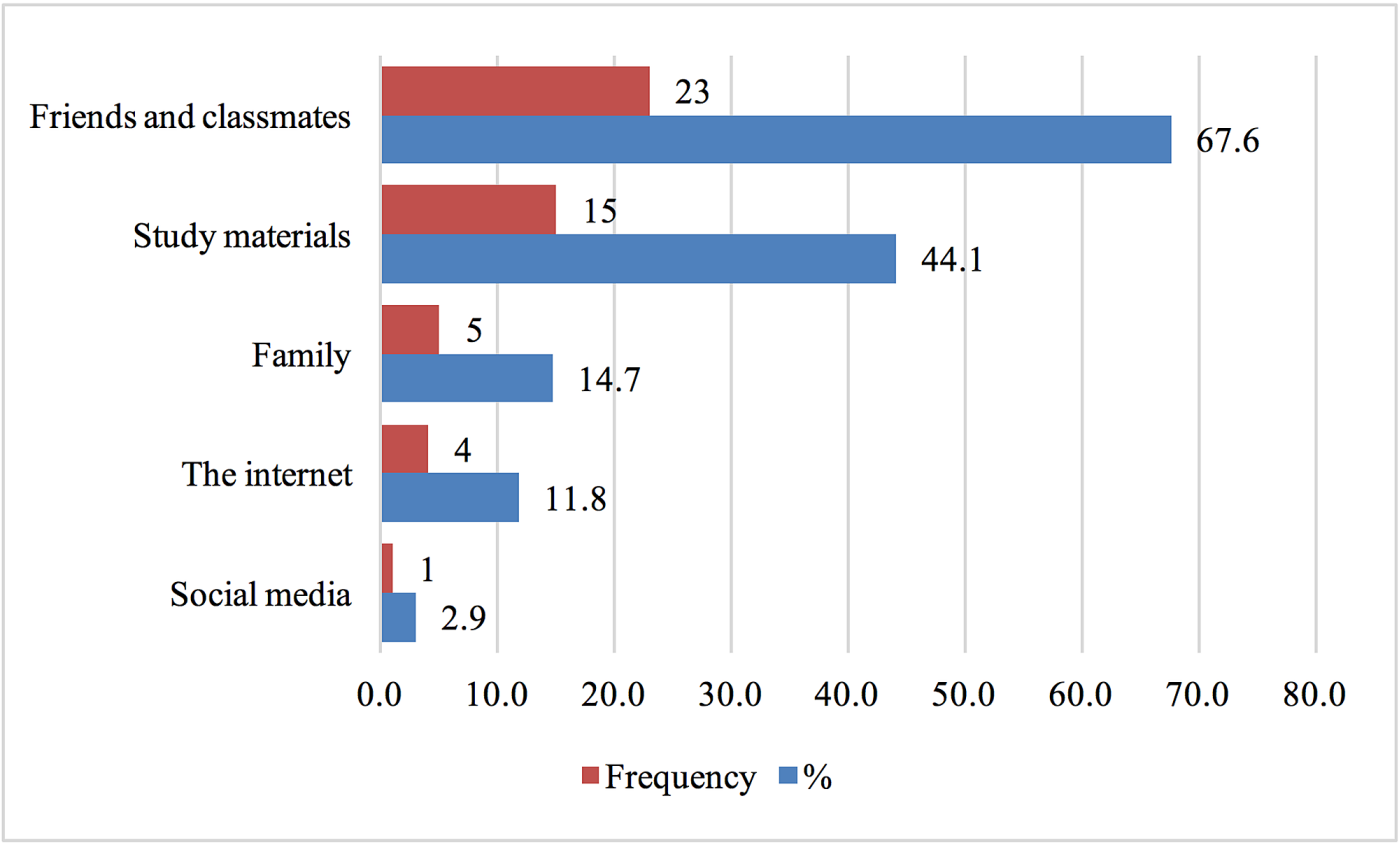
Source of Students' Knowledge of Beta-Blockers

Twenty-three participants (67.6%) out of the 34, learned about propranolol through friends and classmates. The participants had the option of choosing more than one answer for this question; other choices included social media, internet, family, and study materials (Figure 2).

**Figure 3:**
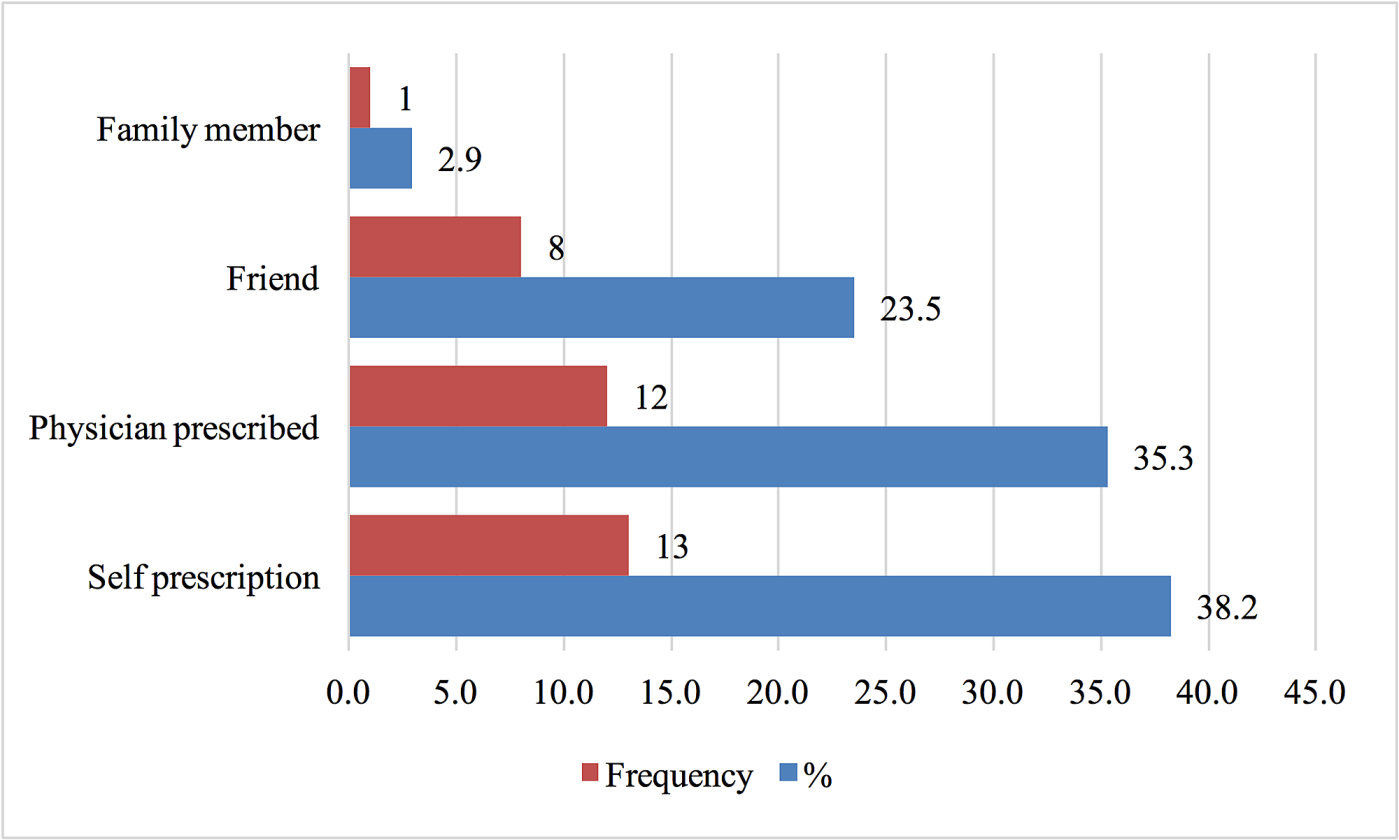
Prescription of Propranolol

When asked about the prescription method, self-prescription ranked first, followed by a physician's prescription (Figure 3). 

The most common frequency of propranolol use was monthly in 19 participants (55.9%), daily in five (14.7%), rarely in six (17.6%), and as needed in four (11.8%). The highest reported dose was 40 mg, almost 50% of the participants consumed 10-20 mg of propranolol as a regular dose. Ten participants (29.4%) increased their propranolol dose without a physician's instructions.

**Figure 4:**
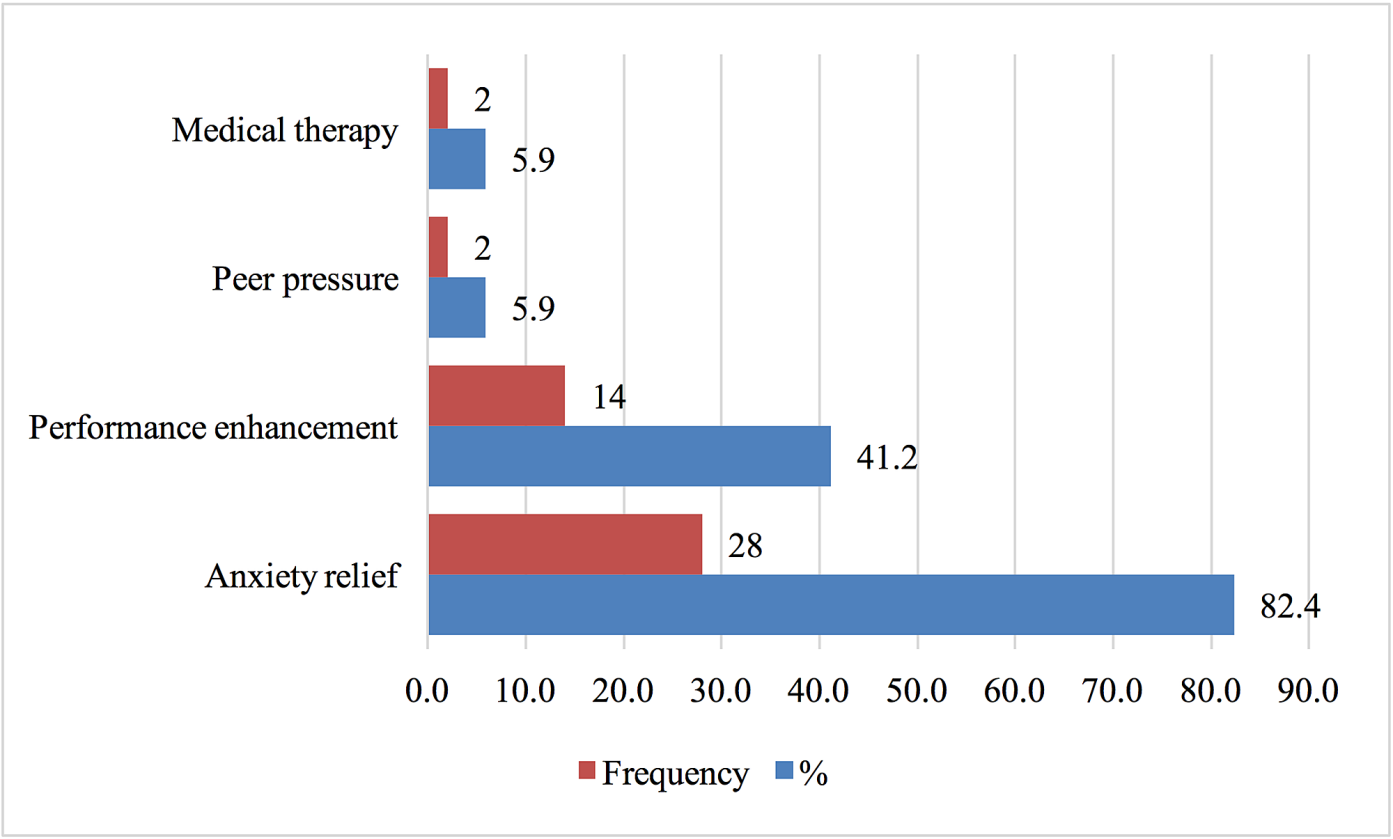
Causes of Using Propranolol

The majority of students reported stress relief as the leading reason behind their propranolol use (82.4%). Other responses included performance enhancement, peer pressure, and medical therapy (Figure 4).

**Figure 5:**
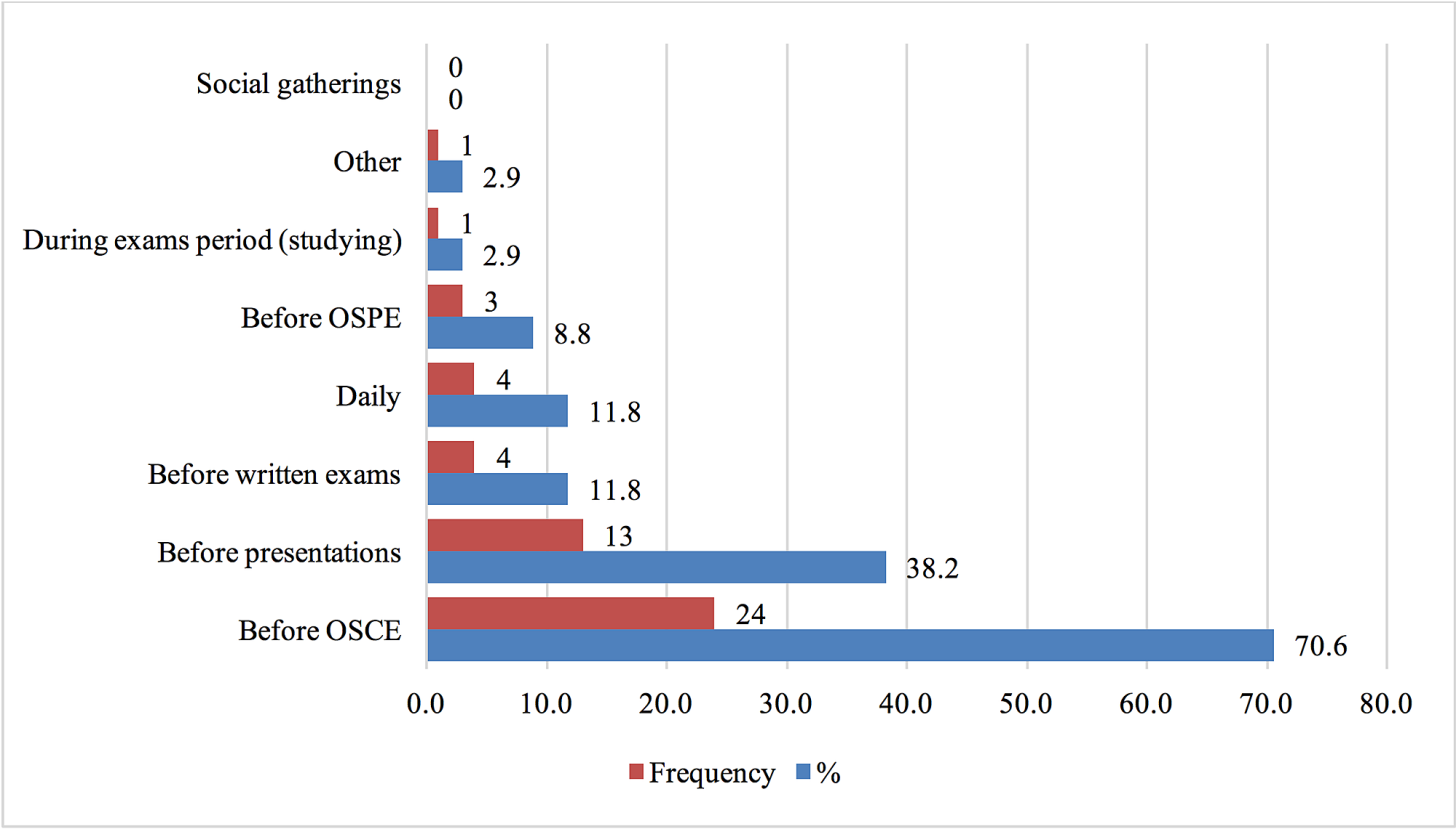
Timing of Propranolol Use OSPE: objective structured practical examination, OSCE: objective structured clinical examination.

The most frequent timing of propranolol use was before OSCEs (n=4, 70.6%; Figure 5). Only four participants (11.8%) reported that they took the drug daily.

**Figure 6:**
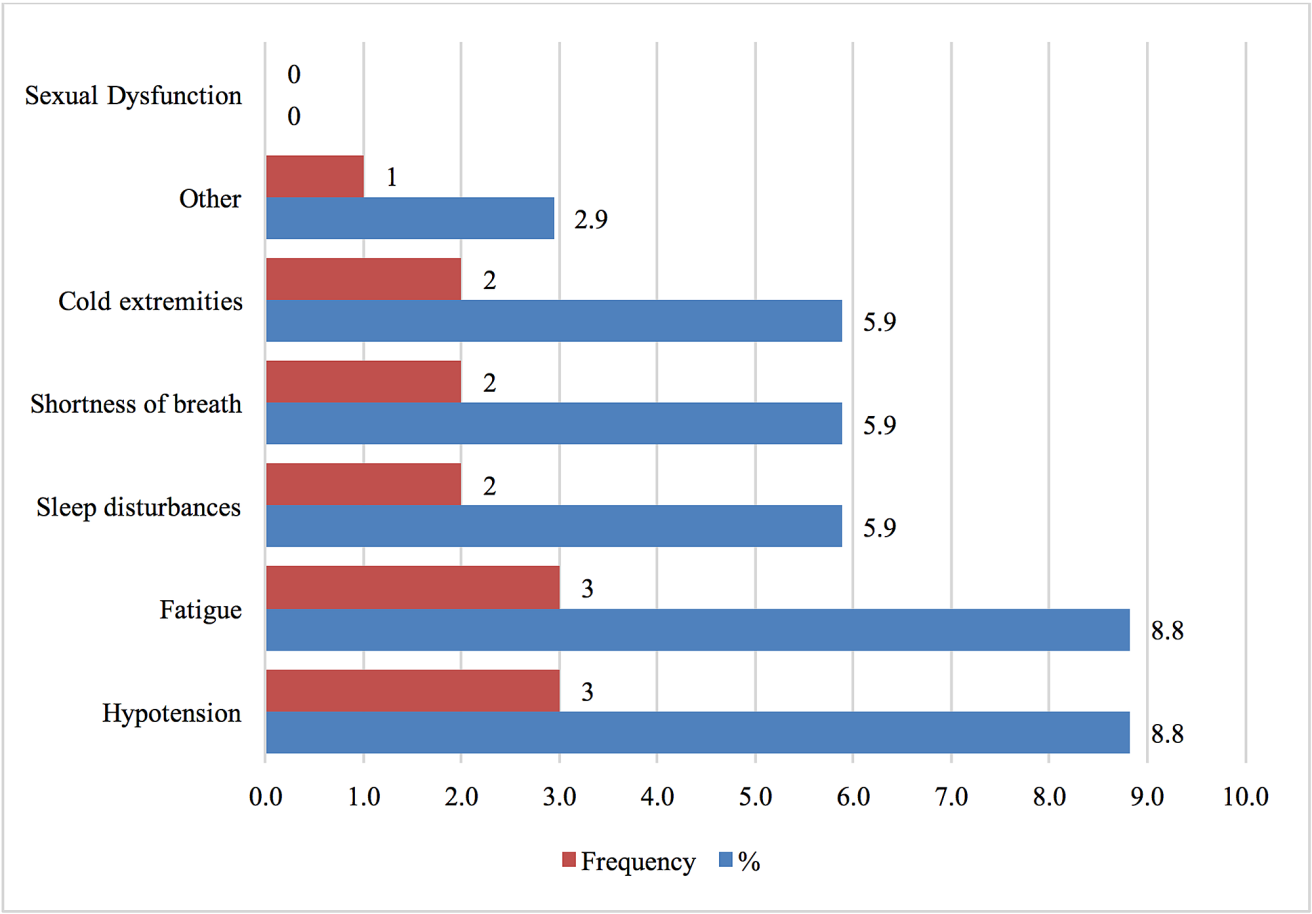
Side Effects Experienced With Using Propranolol

Twenty-seven students (79.4%) were aware of the side effects of unsupervised propranolol use. However, only six students (17.6%) have previously experienced side effects. As demonstrated in Figure 6, students (73.5%) thought that propranolol improved their performance. While 29 (85.3%) participants (17.6%) were recommended propranolol pills by friends and classmates, 20 (58.8%) students reported offering and recommending propranolol to friends and classmates. 

Results of not using propranolol

Of the 200 participants (85.5%) who did not use propranolol, 53 (26.5%) were recommended propranolol, and 31 (15.5%) were offered propranolol.

## Discussion

The prevalence of self-reported propranolol use among the studied sample of medical students at KSAU-HS was 14.4%. Compared to a study conducted among university students in Switzerland [12], the prevalence was higher in our population, and it was found that 1.2% of students used beta-blockers for their psychoactive properties [12]. However, when our findings were compared with a study conducted from Saudi Arabia, it was found that 30% of medical and dental students at KSAU-HS used propranolol during their college years [13]. According to a German study conducted in a general population, 38.8% of people used psychoactive drugs for non-medical reasons such as coping with stress and cognitive performance enhancement [15]. Beta-blockers comprised (8.5%) of the used drugs in that study [15].

It is apparent that the pattern of propranolol use among medical students is higher than the general population. This increased prevalence could be the result of medical students' knowledge of the drug from their study materials, and the varying levels of stress academically and socially. The discrepancies between the reported prevalence percentage of propranolol use among medical students can be attributed to the variation in samples between studies. 

Most propranolol users in the sample reported taking the drug before OSCEs. The setting in the OSCEs may contribute to the anxiety associated with it, as every student is assessed for their skills and knowledge in different clinical scenarios. The numerous exam stations and time limitations per station (seven minutes) places pressure on students. As previously demonstrated in a study on medical students' perceptions of different examination methods, 63% of respondents reported that they find the OSCE very stressful, and 50% thought the time given was not enough [16]. Oral presentations were another occasion where participants reported using propranolol (38%), which like OSCEs, include a social component. However, objective structured practical exams (OSPE; 8.8%) and written exams (11.8%) were minor reasons for propranolol use. Interestingly, half of the students who used propranolol in our study were in their fifth year, which is the year they start the clinical phase. This coincides with the introduction of OSCEs, which constitute a great portion of the grading system in the clinical years. 

Social anxiety is more common among female students than males [17]. This can explain the increased pattern of propranolol use among females, as they used it for performance-enhancement purposes. This study has demonstrated a statistically significant higher propranolol use in females.

In addition to using propranolol, 58.8% have participated actively in recommending or offering it to other students. A considerable percentage of those who do not use propranolol reported being offered or recommended the drug by other students to use it. Such findings suggest the role peer pressure may play in propranolol use among students. It can also be indicative of a behavioral mannerism that normalizes using propranolol.

Drug restriction policies in pharmacies and accessibility vary in different countries, contributing to the varying percentages of use. A study covering six regions of Saudi Arabia found that only 63% of pharmacies adhered strictly to policies regarding prescription-only medications. Seventy-three percent of these pharmacies dispensed propranolol without a prescription, despite the listing of propranolol as a prescription drug by the Saudi Food and Drug Authority [18,19]. Students may decide to use drugs without a physician's assessment or prescription due to easy access through peers or directly from pharmacies. This might explain why only 35.3% of the users in our study had a physician's prescription for propranolol, which raises safety concerns about unsupervised use of this medication. 

This study was limited by a small sample size that consisted of medical students at a single college. Generalizing these findings on the general population is not possible. This can be overcome in the future by conducting further research that includes other colleges and including a wider student population. Another limitation of our findings is the sample skewness, as the participants from various academic levels of college were not equally represented.

## Conclusions

The prevalence of propranolol use among medical students in KSAU-HS Jeddah was low. Fifth-year medical students, in particular, have disproportionately reported the highest rates of propranolol use, accounting for 50% of all users. This increase may be attributed to the development of new stressors during the clinical phase of medical school. Furthermore, anxiety relief for OSCEs and presentations was the main reason for propranolol use. The majority of those who used propranolol were aware of its side effects. Nonetheless, it was most commonly self-prescribed. Educational entities should undertake initiatives towards raising medical students' awareness of the risks of unsupervised propranolol use. Medical students should also be encouraged to seek professional advice before using propranolol.

## Figures and Tables

**Figure 7:**
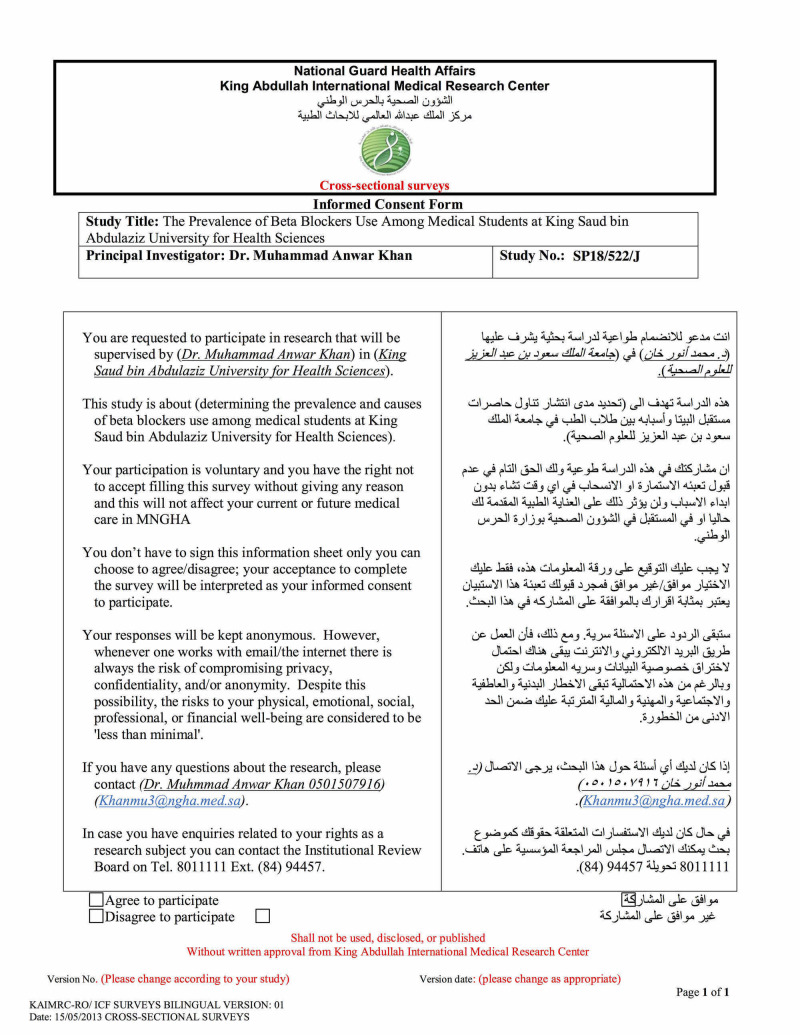
Consent form

## Appendices

The consent form

The questionnaire

**Table 3:** Section 1

Demographics	
College	☐ King Saud bin Abdulaziz University for Health Sciences ☐ King Abdulaziz University ☐ Batterjee Medical College ☐ Ibn Sina National College ☐ Umm Al-Qura University
Academic year	☐ Third-year ☐ Fourth-year ☐ Fifth-year ☐ Sixth-year
Stream	☐ Stream I ☐ Stream II
Gender	☐ Female ☐ Male
Age	
GPA	☐ 5.0 - 4.5 ☐ 4.49 - 4.0 ☐ 3.99 - 3.5 ☐ 3.49 - 3.0 ☐ 2.99 - 2.5
Are you a smoker?	☐ Yes ☐ No
Do you use any illegal or psychoactive drugs?	☐ Yes ☐ No
If yes, what are you using?	
Do you take energy drinks to improve concentration?	☐ Yes ☐ No
During exam week, how many hours do you usually sleep? Hours per day	
Do you exercise?	☐ Yes ☐ No
Are you diagnosed with any chronic diseases?	☐ Yes ☐ No
If yes what is the disease you are suffering from?	
Do you have any allergies or asthma?	☐ Yes, I have asthma ☐ Yes, I have allergies ☐ Yes, I have both ☐ No
Are you diagnosed with any psychiatric disorder?	☐ Yes ☐ No
If yes what is the disorder you are suffering from?	
Do you use beta-blockers/propranolol? (such as Inderal)	☐ Yes skip to question 21 ☐ No skip to question 19

**Table 4:** Section 2

Beta-Blockers Use	
Were you ever recommended beta-blockers/propranolol?	☐ Yes ☐ No
Were you ever offered beta-blockers/propranolol?	☐ Yes ☐ No
END OF QUESTIONNAIRE	

**Table 5:** Section 3

Beta-Blockers Use	
How did you know about beta-blockers? Check all that applies.	☐ Social Media ☐ Internet surfing ☐ Friends and classmates ☐ Family ☐ Study material
Who prescribed propranolol to you?	☐ Prescribed by a physician ☐ Yourself ☐ Friend ☐ Family member
How frequently do you use propranolol?	
What is the dose taken per intake?	☐ < 10 mg ☐ 10 – 20 mg ☐ 30 – 40 mg ☐ > 40 mg
Do you increase the dose without physician instructions?	☐ Yes ☐ No
Why do you use propranolol? Check all that applies.	☐ Performance enhancement ☐ Anxiety relief ☐ Peer pressure ☐ Other
When do you use Propranolol? Check all that applies.	☐ Before written exams ☐ Before OSCE ☐ Before OSPE ☐ Before presentations ☐ During exams period (studying) ☐ Social gatherings ☐ Other
Are you aware of the side effects of the unsupervised use of Propranolol?	☐ Yes ☐ No
If yes, what are the side effects you know? Check all that applies.	☐ Hypotension ☐ Sexual dysfunction ☐ Fatigue ☐ Sleep disturbances ☐ Shortness of breath ☐ Cold extremities ☐ Other
Did you experience any side effects?	☐ Yes ☐ No
If yes, what are the side effects you experienced? Check all that applies	☐ Hypotension ☐ Sexual dysfunction ☐ Fatigue ☐ Sleep disturbances ☐ Shortness of breath ☐ Cold extremities ☐ Other:
Do you think it improves your performance?	☐ Yes ☐ No
Did a friend or a classmate ever recommend propranolol to you?	☐ Yes ☐ No
Did you ever offer propranolol to a friend or classmate?	☐ Yes ☐ No
Would you recommend propranolol to anyone?	☐ Yes ☐ No
END OF QUESTIONNAIRE	
